# A miniaturised method for feeding rate in daphnids–A physiology endpoint for risk assessment

**DOI:** 10.1016/j.namjnl.2025.100009

**Published:** 2025-01-21

**Authors:** Emma Rowan, Flavia Melati Chiappara, Harry Esmonde, Konstantinos Grintzalis

**Affiliations:** a School of Biotechnology, Dublin City University, Republic of Ireland; b Life Sciences Institute, Dublin City University, Republic of Ireland; c School of Mechanical and Manufacturing Engineering, Dublin City University, Republic of Ireland

**Keywords:** *Daphnia magna*, Toxicity, Miniaturization, Algae, Feeding rate, Fluorescence

## Abstract

Pollution is monitored in the ecosystem with methods that aim to capture the presence of pollutants. However, risk assessment is more oriented to develop sensitive metrics that rely on Novel Approach Methodologies (NAMs) which can support existing methods and offer mechanistic insight to the action of pollutants. Feeding is a phenotypic endpoint assessed in many organisms to describe changes in their physiology. For daphnids, feeding is usually quantified with assays employing large volumes and long incubation periods or even cumbersome techniques. In this paper we present a more robust and a faster method to assess feeding with simple fluorescence measurements based on the consumption of algae. The optimized protocol has been tested in a range of pollutants and feeding has proved to be a sensitive indicator for non-lethal exposures.

## 1 Introduction

Human activities result in the release of contaminants into aquatic ecosystems, which threaten wildlife and degrade water quality, thereby making water bodies unsuitable for use (I et al., 2020). Current water monitoring practices typically measure the basic physical and chemical parameters such as pH, temperature, dissolved oxygen and contaminants i.e. nitrates and phosphates (Zolkefli and al., 2020), however, these metrics often miss complex pollutants such as pharmaceuticals, microplastics, and endocrine-disrupting chemicals. In addition, measurements of fauna and flora are also used in spot and grab assessments, but still, they are often too late to identify early pollution before it reaches precarious levels. To address this, New Approach Methodologies (NAMs) for environmental risk assessment have been proposed (Parish et al., 2020).

Daphnids are sentinel species and can serve as early warning systems for freshwater pollution as model organisms. Their short generation time allows for culturing large populations in the lab, and their parthenogenetic life cycle enables the rearing of genetically identical clones eliminating background differences which are useful for ecological and molecular studies. As keystone species in freshwater ecosystems, daphnids are used to set environmental limits on hazardous substances, contribute to chemical risk assessments, and offer the potential for water bioremediation through bioaccumulation or biotransformation of chemicals (Abdullahi et al., 2022).

Current techniques using daphnids as NAMs primarily focus on mortality as an easy indicator of toxicity. However, pollutants can cause harmful effects even at non-lethal levels, necessitating more sensitive sublethal endpoints to understand their underlying toxicity mechanisms (Bownik, 2020). A number of phenotypic endpoints such as swimming, reproduction, growth, feeding, heart rate, and respiration are extensively used as indicators.

Measuring rates of consumption of food (algae) alongside other endpoints offers immediate insights into the physiological effects on daphnids and their environment. While the quantification of the ingestion rate is not a new idea, most of the current methods involve cumbersome processes which make them less accessible. Conventional feeding assays are widely used to evaluate algal consumption in aquatic organisms through various methods, including cell enumeration, fluorescence-based measurements, or radiolabelled algal tracers (Aruoja et al., 2024; Grintzalis et al., 2017). Despite their utility, these approaches present several challenges. Many require the use of older organisms to achieve sufficient ingestion rates, or rely on large assay volumes, and necessitate continuous agitation to prevent algal sedimentation during prolonged incubation periods. Moreover, such assays are often conducted under low-light conditions to inhibit algal proliferation, adding complexity to experimental setups (Hite et al., 2020; Reynaldi et al., 2006). Therefore, there is a need to develop a more efficient and accurate method to measure ingestion rates quickly and reproducibly. Based on previous studies, our group has extensive experience in designing feeding experiments (Grintzalis et al., 2017; Rowan et al., 2024; Giannouli et al., 2023).

This study aimed to explore the use of algae as a standard food source for daphnids. We developed a rapid approach that utilizes small volumes and short incubation periods to measure ingestion. To optimize the protocol, we focused on three key factors; the number of animals used per replicate (well), the duration of the incubation period for feeding, and the volume during incubation. This optimized protocol was then applied to assess the impact of various pollutants on ingestion of daphnids.

## 2 Materials and methods

### 2.1 Materials

All chemicals used in this study were of the highest analytical quality. Diclofenac sodium, trimethoprim, DL-propranolol hydrochloride, dimethylbiguanidine hydrochloride (metformin), 4-acetamidophenol, nickel (II) sulfate, and zinc sulfate were purchased from Acros Organics. Aluminium sulfate hexadecahydrate and lithium chloride anhydrous were purchased from Fisher Scientific. Amoxicillin was purchased from Sigma Aldrich. Glyphosate, 1-ethyl-3-methylimidazolium chloride, and 1-*n*-hexyl-3-methylimidazolium chloride were purchased from ThermoFisher.

### 2.2 Culturing of daphnids and acute toxicity exposures

Daphnids were cultured in aqueous media (final concentrations 0.29 g CaCl_2_·2H_2_O/l, 0.123 g MgSO_4_·7H_2_O/l, 0.065 g NaHCO_3_/l, 0.0058 g KCl/l, 2 μg Na_2_SeO_3_/l, pH 7.7) in a 16h:8 h of light:dark photoperiod at 20 °C, at a density of 80 adults per 4 L media. Media were renewed every six days and cultures were fed daily with algae (*Chlamydomonas rheinharti*) suspension and an organic seaweed extract (*Ascophylum nodosum*) as described previously (Michalaki et al., 2022). For experiments, fifteen neonates (<24 h) from the third brood of their mothers were exposed in a volume of 50 ml for 24 h to each chemical or clear media (used as a negative control to ensure baseline behavior in the absence of chemical stressors) to simulate the exposure for the development of the optimized feeding assay. Toxicity curves were plotted for all chemicals tested with the Hill four-parameter logistic (4PL) model, following the equation Span = Top − Bottom and *Y* = Bottom + (Top-Bottom)/(1 + 10^((LogIC50-X)*HillSlope)), and the parameters top and bottom were commonly fixed to 100 and 0, accordingly, and the model defines in the dotted line the 95 % confidence intervals. EC_50_ values were calculated and a concentration of 1 % and 10 % of EC_50_ was selected for each chemical as non-lethal to assess the impact of each chemical to feeding.

### 2.3 Feeding assay and imaging

The feeding assay was optimized based on the findings detailed in the Results section where key parameters including the algae concentration, the number of animals per well, and the duration of the incubation period, were evaluated to identify the most suitable conditions for feeding. The optimised method which was used to assess the impact of pollutants on feeding requires the following conditions: six daphnids (following exposure for 24 or 48 h to each chemical) were incubated in a 24 well plate with 2.5 ml algae (50,000 cells per ml) and fluorescence was measured (at Ex/Em 560/590 nm using a TECAN plate reader, which had monochromators to select specific wavelengths) in the linear range of consumption after five hours incubation. To calculate the consumption of algae from daphnids, wells with 2.5 ml algae without animals were used as reference. These reference wells provided baseline fluorescence measurements, representing the initial algae concentration in the absence of any feeding activity. The difference in fluorescence was recorded and converted to algae consumed per individual using a corresponding standard curve. To prove that algae was ingested, indicative images of the gut were taken from the animals before and after feeding to show the empty guts of animals before and after feeding.

### 2.4 Statistical analysis

For statistical analysis, data were presented as average ± SD (N = 6). Significant differences were identified first by one-way ANOVA among all exposures as independent groups and then followed by comparisons with the unexposed control using a Dunnett's post-hoc test.

## 3 Results

### 3.1 Optimization of a feeding protocol

**Fig. 1:**
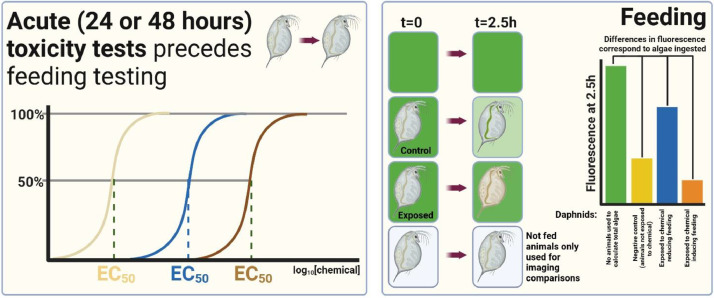
Principle of the feeding assay. Algae is measured as the difference in fluorescence over a period of ingestion of daphnids to feed on algae. Figure created with Biorender.

The principle of the method developed is based on the consumption or ingestion of algae from daphnids and its measurement via fluorescence. As a reference point, wells without animals would be used to account for the difference of fluorescence “missing” from the well and accounted as the ingested algae (Fig. 1).

**Fig. 2:**
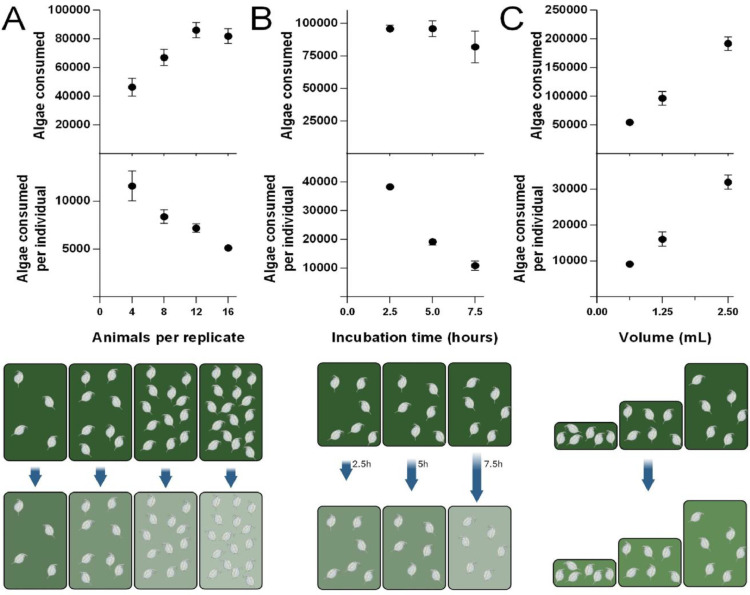
Optimization of the feeding assay. Daphnids were incubated with algae and the difference in fluorescence from wells without animals was converted to algae consumed. Data represents average ± SD (N = 6 replicates for each condition). The animal number per replicate (A), time of feeding incubation (B) and volume (C) were optimized.

Based on previous methods, the time of incubation of daphnids to feed with algae and the number of animals in each well are important parameters of the test (Grintzalis et al., 2017; Rowan et al., 2024; Giannouli et al., 2023). In relation to the number of animals, the more animals per replicate, the more reproducible the test as the differences per individual would be negligible. This was shown with the algae consumed per individual where the spread in wells with four animals per replicate was higher than the rest. However, the increase in the number of animals would also result in an increased “speed” or rate of ingestion. This was observed until a point in a linear fashion between four to eight animals in each well, however, above twelve animals there was a plateau reached, meaning that there is a higher consumption rate than available animals to feed on them (Fig. 2A). To optimize the feeding assay for both time and volume, six animals were placed in wells containing algae, and feeding activity was measured at 2.5-hour intervals. This interval was selected based on preliminary findings, which indicated it provided sufficient time to detect measurable decrease in algae concentration due to feeding. The experiment was repeated using three different volumes, 0.625 ml, 1.25 ml, and 2.5 ml.

Although the algae suspension was consistent at 50,000 cells/ml across all volumes, the absolute quantity of algae varied proportionally with volume, with larger volumes containing greater total algae. Results showed that for incubation periods exceeding five hours, feeding activity began to decrease slightly, suggesting that a maximum incubation period of five hours is optimal for reliable measurement of consumption (Fig. 2B).

The effect of volume on feeding was also evaluated to determine if the test could be scaled down from 2.5 ml to 1.25 ml or 0.625 ml. While the algae concentration remained consistent, the absolute number of algae available for feeding was lower in smaller volumes. As expected, the consumption was reproducible, however, the amount of ingested algae decreased linearly and the preferred approach would be the one with the highest ingestion, the 2.5 ml volume (Fig. 2C). From the above conclusions, the optimized conditions were selected as 2.5 mL of algal suspension of 50,000 cells per ml, and six animals to incubate for 5 h maximum. Representative images of daphnids before and after feeding verify the ingestion of algae being present in the gut of the animals (Supplementary Figure 1).

### 3.2 The impact of pollutants on feeding

The new protocol was tested on a number of pollutants to assess their impact on feeding rates at non-lethal exposure concentrations for pre-exposure at 1 mg/L for 24 h, or at 0.1 mg/L for 48 h which were significantly lower than any mortality observed for the pollutants tested, as shown by the higher EC_50_ values (Table 1). The general trend was a decrease in feeding for all cases of pollutants. Specifically, lithium caused significant reductions of 15 % (24 h) and 27 % (48 h). Nickel, aluminium and zinc had no significant effect at 24 h, but caused a sharp reduction of 57 %, 32 % and 19 % at 48 h, respectively. In relation to pharmaceuticals, diclofenac significantly reduced feeding by 42 % and 18 % at 24 and 48 h, respectively. In a similar manner, metformin reduced feeding by 43 % at 24 h and 15 % at 48 h Propranolol caused the most pronounced reductions, with 51 % at 24 h and 27 % at 48 h Trimethoprim significantly reduced feeding by 33 % at 24 h and 23 % at 48 h and finally, amoxicillin showed no significant changes in feeding at 24 h but it was the only chemical that increased feeding at 48 by 24 %. For ionic liquids, HMI showed no significant changes in feeding at 24 h and decreased feeding by 56 % at 48 h, while EMI had no significant effects at either time point. Finally, glyphosate caused significant decreases by 39 % and 28 % for 24 h and 48 h, respectively.

**Table 1:** The impact of pollutants on the feeding rate of daphnids. Data represents average ± SD (N = 12 replicates for each condition). Statistically significant differences (marked in bold) were identified with one way ANOVA followed by comparison with the control using a Dunnett's post-hoc test. Asterisks indicate statistically significant differences: *p < 0.05, **p < 0.01,***p < 0.001, ****p < 0.0001. Abbreviations: EMI: 1-ethyl-3-methylimidazolium chloride; HMI: 1-n-hexyl-3-methylimidazolium chloride.

Chemical	EC_50_ (in mg/L)	Feeding rate (algae cells consumed per individual for 5 h) for daphnids			
		exposed 24 h to 1 mg/L		exposed 48 h to 0.1 mg/L	
Control	–	10,998±1023	–	9203±1342	–
Lithium	56.86	9356±777	−15 %*	6735±1110	−27 %***
Nickel	69.99	9880±1676	−10 %	3922±686	−57 %****
Aluminium	69.81	10,313±1271	−6 %	6288±912	−32 %****
Zinc	81.86	10,934±1173	−1 %	7499±1197	−19 %**
Diclofenac	43.74	8608±662	−42 %**	7524±592	−18 %*
Metformin	86.14	6291±766	−43 %****	7839±939	−15 %*
Acetaminophen	78.41	8562±977	−22 %**	8470±1062	−8 %
Propranolol	14.24	5345±841	−51 %****	6761±1295	−27 %****
Amoxicillin	-	10,777±1934	−2 %	11,376±1531	+24 %**
Trimethoprim	-	7415±1170	−33 %****	7121±1061	−23 %**
EMI	146	11,948±1595	+9 %	9614±976	+4 %
HMI	1.96	10,693±1565	−3 %	4052±640	−56 %****
Glyphosate	47.19	6678±1138	−39 %****	6650±865	−28 %****

## 4 Discussion

This study assessed the effects of various pollutants on daphnid feeding behaviour, using algae as a food source, while incorporating non-invasive New Approach Methodologies and adhering to the 3Rs principle (Replacement, Reduction, and Refinement). Daphnids, as an important model organism in ecotoxicology, provide a valuable tool for advancing both ethical and scientific standards. Non-invasive endpoints, such as feeding behaviour, capture the live, active status of the physiology of the organism at sub-lethal concentrations. Algae fluorescence was used to assess feeding activity as a method that minimises stress and allows the accurate monitoring of the response of this organism to pollutants.

The optimization of the experimental parameters in this study aimed to improve the accuracy of feeding rate assays for daphnids, aligning with findings from previous research (Grintzalis et al., 2017; Rowan et al., 2024; Giannouli et al., 2023). The results clearly indicate that experimental conditions significantly influence ingestion rates. A linear relationship was observed between the number of animals per replicate and overall algae consumption, with individual feeding rates declining as animal density increased. This suggests that while higher animal densities may accelerate total algae depletion, they could obscure true ingestion rates by reducing per capita food availability. Conversely, lower animal densities led to greater variability, potentially reflecting physiological differences among individuals. Additionally, algae consumption increased with longer incubation times, peaking at around 5 h before stabilizing, indicating a saturation point where ingestion rates level off. As the volume of the medium increased, ingestion rates also increased. While the concentration of algae remained constant, the total number of algae available increased at higher volumes, providing more food (in absolute number of algae in each well) for daphnids to consume. This finding suggests that larger volumes offer a more abundant resource environment, enhancing feeding rates. These results highlight the importance of controlling both animal density, incubation time, and volume to ensure that feeding assays accurately represent natural feeding behaviors in daphnids. These findings in the optimization are in agreement with previous observations also with the consumption of fluorescent microparticles and algae and provide confidence in this new method (Grintzalis et al., 2017; Rowan et al., 2024).

Algae were selected as the food source in this method because they closely mimic the natural dietary components of daphnids, providing ecological relevance to the study. Unlike microparticles, algae are biodegradable and do not introduce synthetic materials into the environment, making them a more environmentally sustainable choice (Cole et al., 2011). Furthermore, using algae avoids the risk of microplastic pollution, which is a growing concern in aquatic ecosystems (Galloway and Lewis, 2016). This approach not only aligns with the principles of environmental responsibility but also enhances the ecological validity of the feeding assay, as algae serve as a high-value, nutrient-appropriate food source for daphnids (Borowitzka, 2013). Research consistently demonstrates that daphnids exhibit a clear preference for algae over synthetic particles such as microplastics when both are present. For example, Isinibilir et al. (2023) (Isinibilir et al., 2023) found that when daphnids were given a choice between algae and polystyrene microplastics, they consumed significantly fewer microplastics in the presence of algae. This suggests that algae availability decreases microplastic ingestion over time, highlighting the preference of daphnids for natural food sources. Similarly, Lan et al. (2024) (Lan et al., 2024) showed that food concentration plays a key role in the ingestion of synthetic particles, with results aligning with those of Isinibilir et al. (2023). In their study, particle consumption was lower when no food was available, increased at medium algae concentrations, and dropped sharply at higher algae concentrations, indicating that daphnids prioritize algae over synthetic particles as food.

The selected pollutants represent a broad range of common contaminants encountered in freshwater ecosystems (Fent et al., 2006; Scheurer et al., 2012; Aral and Vecchio-Sadus, 2008; Kümmerer, 2009; Solomon and Thompson, 2003), providing a realistic perspective on their effects on the ingestion rates of daphnids. Human activities contribute significantly to aquatic pollution, threatening both ecosystems and water quality. Freshwater systems are particularly vulnerable, as they are often contaminated by untreated industrial and urban discharges. Globally, 80 % of municipal wastewater and millions of tons of industrial pollutants, including heavy metals and toxic sludge, enter water bodies each year (I et al., 2020). Agriculture, using 70 % of global water resources, also releases agrochemicals, organic matter, pharmaceuticals, and sediments, further endangering aquatic ecosystems, human health, and economic activities (I et al., 2020).

The observed differences between the 24-hour exposure at 1 mg/L and the 48-hour exposure at 0.1 mg/L can be attributed to the time-dependent and concentration-dependent toxicity mechanisms. Longer exposure times often amplify the bioaccumulation of chemicals, increasing their physiological impact, which can be observed even at lower concentrations. This is especially true for chemicals that have delayed toxicity pathways, where effects such as oxidative stress or enzyme inhibition intensify with prolonged exposure. Additionally, the higher initial concentration (1 mg/L) in the 24-hour exposure may lead to acute but less pronounced effects compared to the cumulative damage caused by 48-hour exposure at 0.1 mg/L. This aligns with findings in ecotoxicology where sublethal stress during extended exposure can reduce energy reserves, affecting behaviours like feeding rates. Environmental factors like chemical solubility and bioavailability over time also play a role in modulating these effects (Raimondo et al., 2009; Cairns and Pratt, 1988).

To the best of our knowledge, this study is the first to investigate the effects of these specific pollutants using a feeding assay in which algae serve as the food source for daphnids, measuring either an inhibition or an increase in their feeding rate. Since there are no directly comparable studies in literature, this represents a novel approach to assessing the impact of these pollutants. However, research on similar compounds in aquatic systems provides some contextual insights, even though differences in organisms and methodologies limit direct comparisons. Previous feeding protocols, such as those using fluorescent microparticles, algae cell counts, or even using optical density to measure changes in algal concentration have primarily focused on alternative experimental techniques and methods (Grintzalis et al., 2017; Rowan et al., 2024; Giannouli et al., 2023; Barata et al., 2008). While these approaches have provided valuable insights into feeding behaviours and pollutant impacts, they differ significantly in sensitivity, ecological relevance, or practical application. This new miniaturised feeding assay offers a unique perspective on the potential ecotoxicological effects of pollutants on daphnids, contributing to an important gap in the understanding of their environmental impact. Revolutionising risk assessment requires such as the development of all-in-one toxicology platforms, which can fit in miniaturised (or box) tools and integrate automation in measurements to from larger exposures as such proposed by many EU consortia including the PrecisionTox (26) PANBioRA and ToxBox. Future research could explore similar assays across different species to further validate and expand upon our findings.

## Supplementary data

**Supplementary Figure 1.** Indicative image of animals before and after incubation with algae. Algae are located in the gut of animals (arrows) after feeding.

## CRediT authorship contribution statement

**Emma Rowan:** Writing – review & editing, Writing – original draft, Visualization, Methodology, Investigation, Formal analysis. **Flavia Melati Chiappara:** Writing – original draft, Methodology, Investigation, Formal analysis, Conceptualization. **Harry Esmonde:** Writing – review & editing, Supervision, Project administration, Funding acquisition, Conceptualization. **Konstantinos Grintzalis:** Writing – review & editing, Writing – original draft, Visualization, Supervision, Project administration, Methodology, Investigation, Funding acquisition, Conceptualization.

## Declaration of competing interest

The authors declare the following financial interests/personal relationships which may be considered as potential competing interests: Harry Esmonde reports financial support was provided by European Union. If there are other authors, they declare that they have no known competing financial interests or personal relationships that could have appeared to influence the work reported in this paper. Konstantinos Grintzalis reports financial support was provided by Taighde Éireann – Research Ireland. If there are other authors, they declare that they have no known competing financial interests or personal relationships that could have appeared to influence the work reported in this paper.

## Data Availability

Data will be made available on request.

## Appendix Supplementary materials

image 1
