# Supplementary figures and images for: A miniaturised method for feeding rate in daphnids–A physiology endpoint for risk assessment
Source: NAM J. 2025 Jan 21;1:100009. doi: 10.1016/j.namjnl.2025.100009 (PMC13289036; doi:10.1016/j.namjnl.2025.100009)

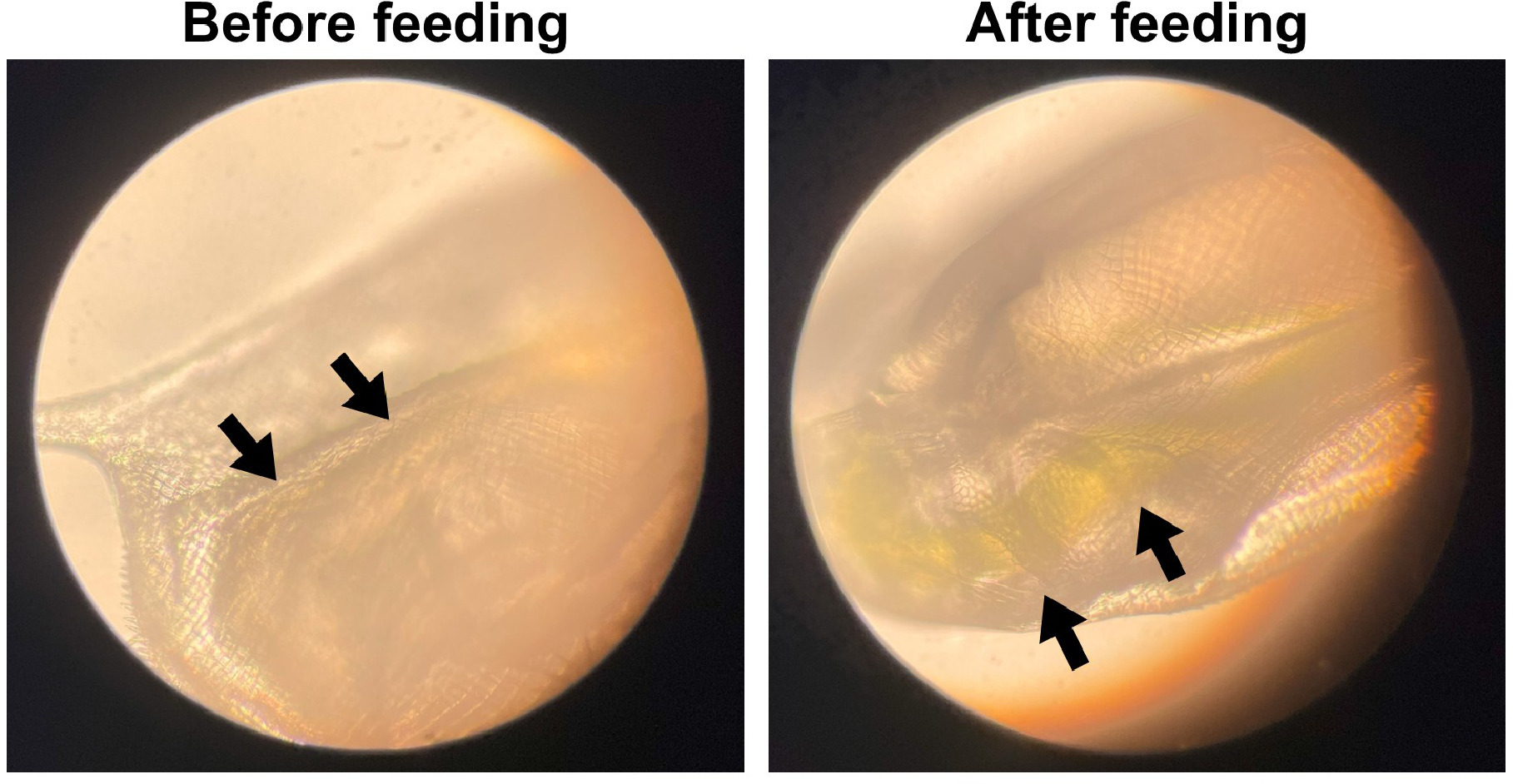

Supplement: Supplementary file 1 [file mmc1.jpg]
